# A nomogram predicting the severity of COVID-19 based on initial clinical and radiologic characteristics

**DOI:** 10.2217/fvl-2020-0193

**Published:** 2022-02-21

**Authors:** Hanfei Zhang, Feiyang Zhong, Binchen Wang, Meiyan Liao

**Affiliations:** ^1^Department of Radiology, Zhongnan Hospital of Wuhan University, Wuhan, 430071, China

**Keywords:** clinical characteristics, COVID-19, nomogram, radiological characteristics

## Abstract

**Aim:** This study aimed to build an easy-to-use nomogram to predict the severity of COVID-19. **Patients & methods:** From December 2019 to January 2020, patients confirmed with COVID-19 in our hospital were enrolled. The initial clinical and radiological characteristics were extracted. Univariate and multivariate logistic regression were used to identify variables for the nomogram. **Results:** In total, 104 patients were included. Based on statistical analysis, age, levels of neutrophil count, creatinine, procalcitonin and numbers of involved lung segments were identified for nomogram. The area under the curve was 0.939 (95% CI: 0.893–0.984). The calibration curve showed good agreement between prediction of nomogram and observation in the primary cohort. **Conclusion:** An easy-to-use nomogram with great discrimination was built to predict the severity of COVID-19.

In December 2019, an outbreak of an unexplained pneumonia occurred in Wuhan city, Hubei province of China. Deep sequencing analysis from lower respiratory tract samples indicated a novel coronavirus, which was named 2019 novel coronavirus (2019-nCoV) [1,2], and was renamed as severe acute respiratory syndrome coronavirus 2 (SARS-CoV-2). The disease caused by SARS-CoV-2 was named COVID-19. The SARS-CoV-2 belongs to the beta coronaviruses, which can cause severe and potentially fatal respiratory tract infection. SARS-CoV-2 exhibits the closest links with two SARS-like coronaviruses from bats (bat-SL-CoVZX45 and bat-SL-CoVZX21) [1].

As of 17 April 2021, there have been 139,501,934 confirmed COVID-19 cases, including 2,992,193 deaths [3]. The clinical features of COVID-19 are similar to previous beta coronavirus infections [4]. The most common symptoms of COVID-19 were fever, cough, myalgia or fatigue; the less common symptoms were sputum production, headache, hemoptysis and diarrhea [4–6]. The typical computed tomography (CT) features of COVID-19 were ground-glass opacity (GGO), intralocular or interlobular septal thickening, located close to visceral pleural surfaces and a multifocal bilateral distribution [7–9]. These typical CT features and clinical features can help diagnose COVID-19. Besides, many models based on deep-learning algorithms also contribute to the diagnosis of COVID-19 [10–13].

Although most patients with COVID-19 are of the common type that have mild symptoms and a good prognosis, COVID-19 can also develop to a severe or critical type where patients need intensive care, mechanical ventilation or reach the end point of death [4,6,14]. Patients with different clinical types require different treatments and care plans [15]. Therefore, it is important to predict the severity of COVID-19 on admission, to help clinicians to decide on treatment plans and prepare for a serious condition in advance. Many studies have reported the differences in clinical and CT features between common-type and severe-type COVID-19 [6,16]. However, only few studies have reported a model that can be easily used clinically to predict the severity of COVID-19. Thus, the aim of this study was to build a model that can be easily used to predict the severity of COVID-19 based on different clinical and radiological characteristics of severe/critical and common types of COVID-19.

## Methods

### Study design & participants

From 31 December 2019 to 22 January 2020, all consecutive patients admitted to the Zhongnan Hospital of Wuhan University and confirmed with COVID-19, were enrolled. Those who were admitted to the intensive care unit that required mechanical ventilation or had a fraction of inspired oxygen concentration of at least 60% or more, were classified as severe/critical type [9]. The other patients were classified as common types. All patients were followed-up until hospital discharge, death or the last follow-up date of 29 February 2021.

### Data collection

The clinical characteristics including demographic data, medical history, epidemiological characteristics, underlying co-morbidities, clinical symptoms and signs, and laboratory findings were extracted from electronic medical records. Two investigators independently reviewed the data collection forms to verify data accuracy. The laboratory tests consisted of complete blood count, coagulation function, liver and renal function, C-reactive protein (CRP), erythrocyte sedimentation rate, procalcitonin, lactate dehydrogenase and creatine kinase.

All CT images were independently reviewed by two experienced chest radiologists (H Zhang and F Zhong) on an image archiving and communication system. Decisions were reached by consensus. Lesion density was classified as GGO, defined as increased lung parenchymal attenuation that did not obscure the underlying vascular architecture; and consolidation was defined as opacification in which the underlying vasculature was obscured [17]. Lesion location was classified as peripheral if it was in the outer a third of the lung; otherwise, it was defined as dispersed. Lesion size was defined as the largest size of lesions and was classified as small (diameter: <1 cm), medium (diameter: 1–3 cm), large (diameter: 3 cm–50% of the segment) or segmental (50–100% of the segment) [18]. The number of involved lung segments was recorded. Other CT features such as intralobular and interlobular septal thickening, air bronchogram, pleural effusion and a short axis diameter of mediastinal lymph nodes larger than 1 cm were also recorded.

### Statistical analysis

Categorical variables were described by frequency rates and percentages; and compared between severe/critical and common type COVID-19 using the χ^2^ test or Fisher’s exact test. A two-sided α of less than 0.05 was considered statistically significant and included in the multivariate logistic regression analysis. Likelihood ratio multivariate logistic regression analysis was used to identify predictors of severity of COVID-19. If not otherwise specified, the variables with p < 0.2 were selected into a relatively parsimonious model as the final model. The above statistical analyses were performed using the SPSS software, version 21.0 for windows (SPSS Inc).

A nomogram was built based on the results of the logistic regression analysis by using R, version 3.6.3 with the rms statistical packages. The area under the curve (AUC) was used to evaluate the performance of the model to predict the risk of severity transformation. The closer the AUC is to 1, the closer the model is to reality. Moreover, during the internal validation of the nomogram, a calibration curve was plotted based on 1000 bootstrap resampling to assess the predictive accuracy of the nomogram [19]. The closer the calibration curve is to the 45-degree diagonal line, the better the performance of the prediction model.

## Results

In our hospital, 133 consecutive patients were confirmed with COVID-19 by pharyngeal swabs samples for real-time RT-PCR. Seventeen patients without chest CT examination within 3 days of admission and 12 nonhospitalized patients were excluded. Eventually, 104 patients with chest CT scans were included in our study. All patients lived in Wuhan, China. Chest CT scans were performed on four different CT machines: 74 on a Revolution HD (GE Healthcare, WI, USA), seven patients on an Ingenuity (Philips Healthcare, MA, USA); 15 on a Somatom Definition (Siemens, Erlangen, Germany) and eight patients on a uCT 750 (United Imaging, Shanghai, China,). The slice thickness of CT images was 1 mm, and CT images were reviewed on lung window (window level -440, window width 1500) and mediastinal window (window level 40, window width 400).

### Clinical characteristics

The clinical characteristics of included cases are shown in Table 1. The study included 54 (51.92%) male patients and 50 (48.08%) female patients. The median age was 54 years old (range: 22–92 years old). Seventy-three (70.19%) patients were common type, 31 (29.81%) patients were severe/critical type. Sixteen (15.38%) patients with severe/critical type passed away; the others were cured and discharged from the hospital. Thirty-nine (31.0%) patients had co-morbidities, including eight (7.7%) chronic pulmonary diseases, ten (9.6%) heart diseases, ten (9.6%) diabetes, 23 (22.1%) hypertension, seven (6.7%) tumor, six (5.8%) cerebral infarction, four (3.8%) gout, three (2.9%) chronic kidney disease and one (1.0%) hyperlipidemia. Of these 39 patients, 23 (58.97%) patients were severe type, and 16 (41.03%) patients were common type. The most common symptoms were fever (83.65%) and cough (54.81%), the less common symptoms were myalgia (21.15%), diarrhea (8.65%) and dyspnea (3.85%). Fifty-one (49.04%) patients had normal leukocyte count, 75 (72.12%) patients had decreased lymphocyte count, 69 (66.35%) patients had normal level of neutrophil count, 64 (61.54%) patients had increased prothrombin times, 65 (62.50%) patients had increased CRP and 62 (50.62%) patients had abnormal albumin levels.

**Table 1.** Clinical and radiologic characteristics and univariable analysis of the risks of severe/critical type of COVID-19.

Variables	Total n = 104 (%)	Severe/critical type n = 31 (%)	Common type n = 73 (%)	p-value
Age (years) <40 40–59 >59	29 (27.9) 30 (28.8) 45 (43.3)	1 (3.2) 4 (12.9) 26 (83.9)	28 (38.4) 26 (35.6) 19 (26.0)	<0.001
Gender Male Female	54 (51.9) 50 (48.1)	18 (58.1) 13 (41.9)	36 (49.3) 37 (50.7)	0.414
Comorbidity	39 (37.5)	23 (74.2)	16 (21.9)	<0.001
Fever	87 (83.7)	24 (77.4)	63 (86.3)	0.263
Cough	57 (54.8)	17 (54.8)	40 (54.8)	0.997
Myalgia	22 (21.2)	7 (22.6)	15 (20.5)	0.816
Diarrhea	9 (8.7)	5 (16.1)	4 (5.5)	0.122
Dyspnea	4 (3.8)	3 (9.7)	1 (1.4)	0.078
Leukocyte count (× 10^9^/l) Increase Decrease	8 (7.7) 45 (43.3)	6 (19.4) 10 (32.3)	2 (2.7) 35 (47.9)	0.011
Lymphocyte count (× 10^9^/l) Decrease	75 (72.1)	27 (87.1)	48 (65.8)	0.032
Neutrophil count (× 10^9^/l) Increase Decrease	10 (9.6) 25 (24.0)	8 (25.8) 3 (9.7)	2 (2.7) 22 (30.1)	<0.001
Prothrombin time (s) Abnormal	64 (61.5)	18 (58.1)	46 (63.0)	0.635
d-dimer (mg/l) Abnormal	15 (14.4)	5 (16.1)	10 (13.7)	0.747
ESR (mm/h) Abnormal	42 (40.4)	14 (45.2)	28 (38.4)	0.518
CRP (mg/l) Abnormal	65 (62.5)	19 (61.3)	46 (63.0)	0.868
Procalcitonin (ng/ml) Abnormal	34 (32.7)	20 (64.5)	14 (19.2)	<0.001
AST (U/l) Abnormal	37 (35.6)	20 (64.5)	17 (23.3)	<0.001
LDH (U/l) Abnormal	40 (38.5)	20 (64.5)	20 (27.4)	<0.001
CK (U/l) Abnormal	20 (19.2)	7 (22.6)	13 (17.8)	0.572
Albumin (g/l) Abnormal	62 (59.6)	26 (83.9)	36 (49.3)	0.001
Creatinine Abnormal	22 (21.2)	12 (38.7)	10 (13.7)	0.004
Lesion size <1 cm 1–3 cm 3 cm–50% of segment ≥50% of segment	6 (5.8) 21 (20.2) 48 (46.2) 29 (27.9)	0 2 (6.5) 11 (35.5) 18 (58.1)	6 (8.2) 19 (26.0) 37 (50.7) 11 (15.1)	<0.001
Number of involved lung segments ≥9 <9	54 (51.9) 50 (48.1)	26 (83.9) 5 (16.1)	28 (38.4) 45 (61.6)	<0.001
Lesion density GGO Consolidation	93 (89.4) 11 (10.6)	29 (93.5) 2 (6.5)	64 (87.7) 9 (12.3)	0.499
Lesion location Peripheral Dispersed	80 (76.9) 24 (23.1)	24 (77.4) 7 (22.6)	56 (76.7) 17 (23.3)	0.938
Intralobular or interlobular septal thickening	16 (15.4)	7 (22.6)	9 (12.3)	0.185
Air bronchogram	46 (44.2)	18 (58.1)	28 (38.4)	0.064

AST: Aspartate aminotransferase; CK: Creatine kinase; CRP: C-reaction protein; ESR: Erythrocyte sedimentation rate; GGO: Ground-glass opacity; LDH: Lactic dehydrogenase.

### Chest CT characteristics

**Figure 1.**
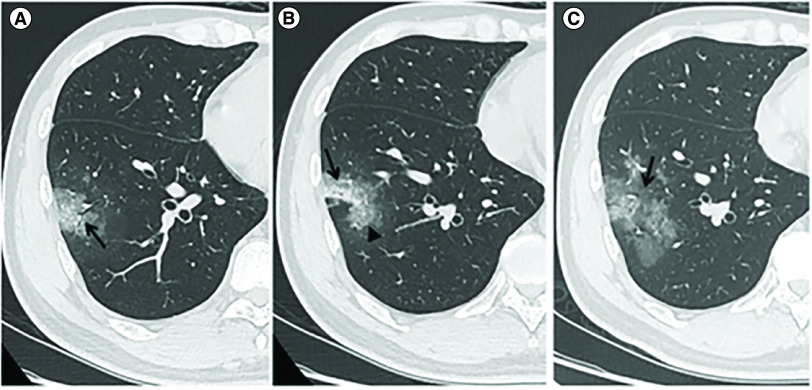
Computed tomography imaging of a case of novel coronavirus pneumonia. **(A–C)** Chest CT of a 37-year-old male who had a fever for 4 days and showed a common type of novel coronavirus pneumonia. **(A & B)** Images of the lung window show an irregular shape of GGO combined with a little consolidation opacity located in the lateral basal segment (S9) of the right lower lobe (arrow). Intralobular septal thickening (arrowhead) and air bronchogram sign (arrow) were also present. **(C)** The follow-up CT image of the lung window 2 days later shows that the density in the middle of the lesion was decreased, but the size of the lesion was increased (arrow). CT: Computed tomography; GGO: Ground-glass opacity.

The CT features of the included cases are shown in Table 1. The density of 93 (89.42%) cases was GGO, 11 (10.58%) cases showed consolidation. The lesion size of 48 (46.15%) cases was 3 cm–50% of segment, 29 (27.88%) cases were ≥50% of segment. Eighty (76.92%) cases showed lesions located peripherally, 16 (15.38%) cases showed intralobular or interlobular septal thickening, 28 (26.93%) cases showed air bronchogram. Figure 1 shows the typical chest CT findings of a COVID-19 patient. One patient with chronic lymphocytic leukemia and one patient with lung cancer had enlarged mediastinal lymph nodes and little pleural effusion. Two patients with chronic obstructive pulmonary disease had enlarged mediastinal lymph nodes.

### Selected factors for nomogram

As shown in Table 1, univariable analysis showed that age (p < 0.001), comorbidity (p < 0.001), leukocyte count (p = 0.011), lymphocyte count (p = 0.026), neutrophil count (p < 0.001), procalcitonin (p < 0.001), aspartate transaminase (p < 0.001), lactate dehydrogenase (p < 0.001), albumin (p = 0.001), creatinine (p = 0.004), lesion size (p < 0.001) and number of involved lung segments (p < 0.001) were significantly related to the severity of COVID-19. As shown in the multivariable logistic analysis (Table 2), age (p = 0.009), neutrophil count (p = 0.141), creatinine (p = 0.033) and number of involved lung segments (p = 0.058) were independent risk factors of severe/critical type of COVID-19. In the multivariable logistic analysis, procalcitonin (p = 0.308) was not significantly related to the severity of COVID-19. However, in consideration of serum concentrations, a high level of procalcitonin was found to be correlated to the severity of microbial invasion [20]. Therefore, procalcitonin was also selected to build the prediction model.

**Table 2.** Multivariable logistic regression analysis of the risk of severe/critical type of COVID-19.

Variables	OR	Multivariable analysis		OR	Selected factors for model	
		95% CI	p-value		95% CI	p-value
Age (years)			0.009			0.001
<40	Ref.					
40–59	104.143	2.033–5335.013	0.021	91.579	3.663–2289.867	0.006
≥60	5.178	0.104–257.531	0.409	5.833	0.240–141.873	0.279
Comorbidity			0.295			
No	Ref.					
Yes	2.331	0.478–11.369				
Leukocyte count (×10^9^/l)			0.578			
Normal	Ref.					
Decrease	1.087	0.127–9.332	0.940			
Increase	0.119	0.002–7.106	0.307			
Lymphocyte count (×10^9^/l)			0.764			
Normal	Ref.					
Abnormal	1.398	0.157–12.483				
Neutrophil count (×10^9^/l)			0.141			0.143
Normal	Ref.					
Decrease	0.664	0.041–10.881	0.774	0.967	0.170–5.496	0.969
Increase	45.294	0.957–2142.909	0.053	9.574	0.951–96.403	0.055
Procalcitonin (ng/ml)			0.308			0.061
Normal	Ref.					
Abnormal	2.592	0.415–16.167		3.873	0.942–15.924	
AST (U/l)			0.300			
Normal	Ref.					
Abnormal	3.029	0.373–24.610				
LDH (U/l)			0.877			
Normal	Ref.					
Abnormal	0.859	0.126–5.870				
Albumin (g/l)			0.595			
Normal	Ref.					
Abnormal	0.557	0.065–4.814				
Creatinine			0.058			0.036
Normal	Ref.					
Abnormal	7.064	0.940–53.111		6.224	1.126–34.403	
Lesion size			0.596			
<1 cm	Ref.					
1–3 cm	60,836,874.90	0.000-	0.999			
3 cm–50% of segment	183,726,613.1	0.000-	0.999			
≥50% of segment	48,424,235.74	0.000-	0.999			
No. of involved lung segments			0.039			0.006
<9	Ref.					
≥9	8.848	1.114–70.276		7.712	1.821–32.667	

AST: Aspartate aminotransferase; LDH: Lactic dehydrogenase; OR: Odds ratio.

### Nomogram building & validation

**Figure 2.**
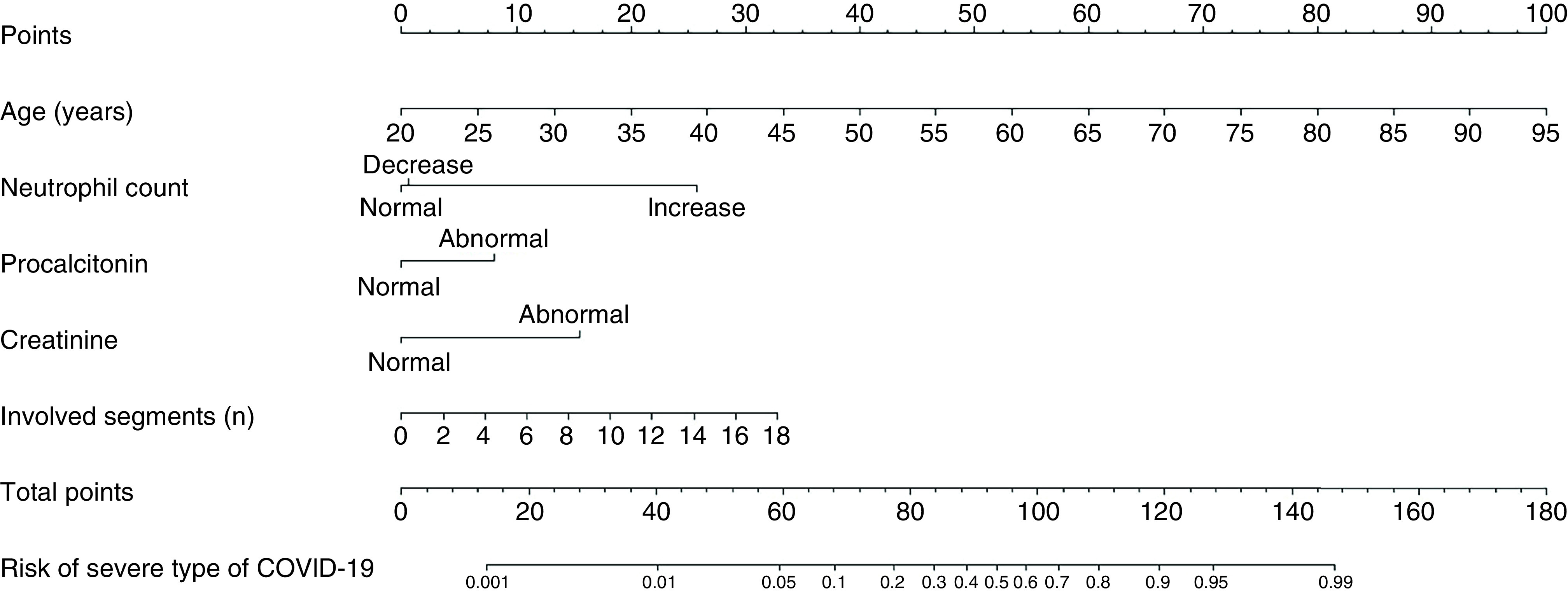
The nomogram predicting the risk of severity of COVID-19. Each variable was assigned a score on the point scale axis. The total score was obtained by adding every single score. Based on the total point scale, we were able to estimate the severity of COVID-19.

Based on the results of regression analysis and clinical consideration, a nomogram was built that incorporated five variables (age, neutrophil count, creatinine, number of involved lung segments and procalcitonin) to predict the severity of COVID-19 (Figure 2). A total score was calculated by adding every single score from the independent five variables. By projecting the total score to the lowest scale, we were able to estimate the severity of COVID-19.

**Figure 3.**
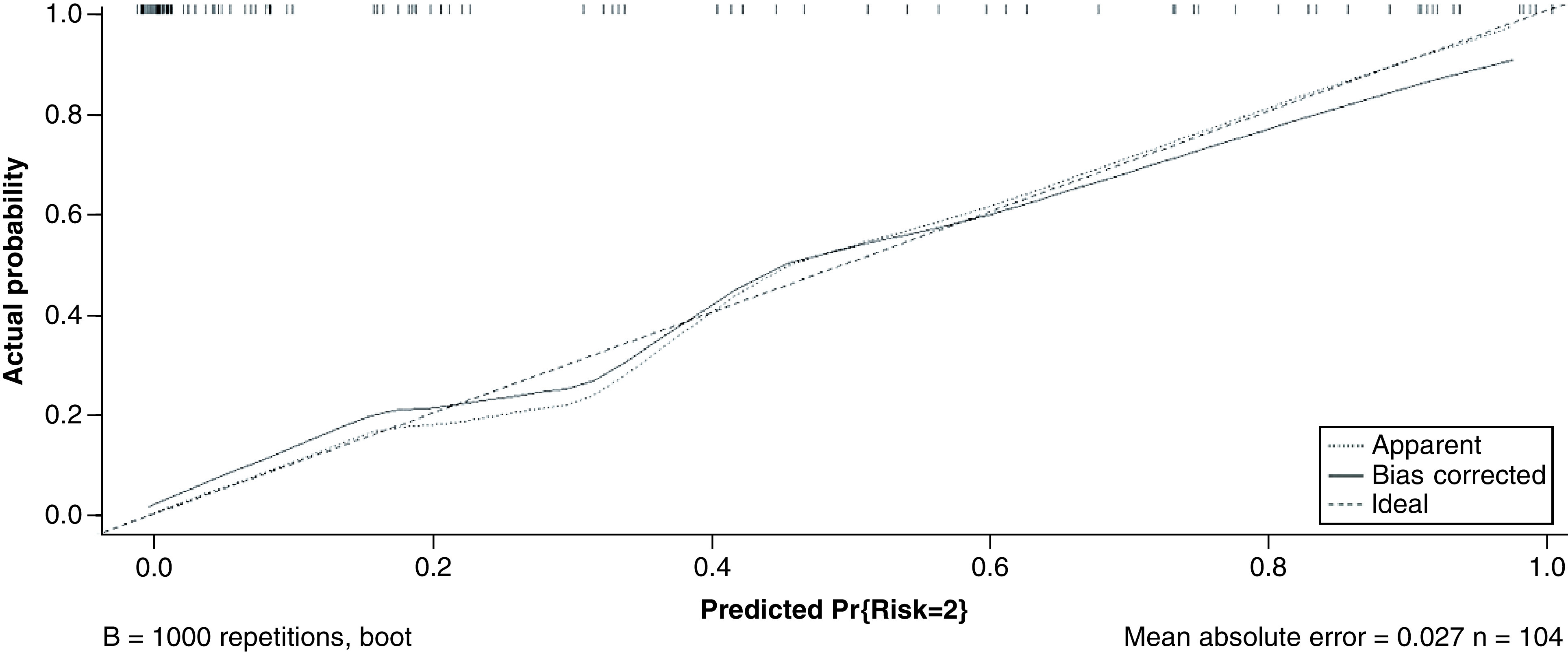
The calibration curves for the nomogram. The horizontal axis is the nomogram-predicted probability, and the vertical axis is the actual probability of a severe type of COVID. The 45° dashed line is the reference line. The dotted line shows the performance of the current nomogram, and the solid line is bias-corrected by bootstrapping.

The AUC was 0.939 (95% CI: 0.893–0.984) in the training set before the bootstrap technique was applied, showing good discrimination of the severity of COVID-19. The calibration curve showed a good agreement between the prediction of the nomogram and the observations in the primary cohort (Figure 3).

## Discussion

The COVID-19 pandemic has become a global public health issue, threatening millions of lives worldwide. As different clinical types of COVID-19 require different treatments and care plans, and the mortality of severely ill patients with COVID-19 is considerable [14,15], it is of vital importance to find a way to predict the risk of disease progression. Thus, in this study, we compared the differences in clinical and radiological characteristics in severe/critical and common type COVID-19 patients, and reported a simple and easily available nomogram, based on the initial clinical and radiological characteristics, to predict the severity of COVID-19. The nomogram incorporates five variables (age, neutrophil count, creatinine, procalcitonin and number of involved lung segments) and has a high discrimination rate of 93.9%.

Nguyen *et al.* [21] reported a nomogram that only included clinical characteristics (age, respiratory rate, overweight, temperature, CRP, troponin and lymphocyte counts) and the C-statistics of their final model is 0.75. Yu *et al.* [22] reported a nomogram that only included age and CT features (density, mosaic perfusion sign and severity score of the lung) to predict the severity of COVID-19 and the AUC of the model was 0.929. In our study, both clinical characteristics (age, neutrophil count, creatinine and procalcitonin) and CT features (number of involved lung segments) were included to build the predictive model, with an AUC of 0.939. Other studies also reported models based on deep-learning algorithms using CT images to predict the severity of COVID-19 and showed a great accuracy [23–25]. However, these models were more difficult to build because they need complicated algorithms, lesion annotation for each object to guide learning of the algorithm or an artificial intelligence-assisted method to label information. Hence, these models are not available for every institution and clinician. In our study, only the original clinical characteristics (age, neutrophil count, creatinine and procalcitonin) and chest CT characteristic (numbers of involved lung segments), which were easily available and identified in clinical, were needed for the predictive mode. The nomogram was a reliable tool that can create a simple intuitive graph of a statistical predictive model that quantifies the risk of a clinical event [26]. Thus, the model built by us was more user-friendly and with a high discrimination rate.

As reported by previous studies, the most common symptoms of COVID-19 were fever and cough, and the most common chest CT characteristics were GGO [4,6,27]. By univariable analysis, we found that age, co-morbidities, leukocyte count, lymphocyte count, neutrophil count, procalcitonin, aspartate transaminase, lactate dehydrogenase, albumin, creatinine, lesion size and number of involved lung segments were related to the severity of COVID-19, which also had been reported before [4,6,27]. The results showed that patients with severe clinical type may have impaired liver and renal functions, activating the body’s inflammatory response. Lymphocytopenia was a prominent feature of severe clinical type, which may be caused by necrosis or apoptosis of lymphocytes; this has also been found in Middle East respiratory syndrome and severe acute respiratory syndromes [28,29]. Men more frequently developed the severe clinical type, but the difference was not significant [14]. Except for dyspnea, no other clinical symptom was significantly related to the severity of COVID-19, which made the discrimination more difficult. In the cases that involved more lung segments initially, the virus was more widely distributed in the lungs and these patients were more likely developed to severe-type cases. The larger the lesion, the larger the extent of the lung was involved, and the more likely the case developed to severe/critical type.

After multivariable analysis, we found that old age, increased neutrophil count, increased creatinine and involvement of more lung segments were independent risk factors of severe/critical COVID-19. An elevated serum procalcitonin level was correlated to the severity of microbial invasion and usually occurred in severe shock, systemic inflammatory response syndrome and multiple organ dysfunction syndrome [20]. Considering this, procalcitonin was added to build the nomogram. Because it was recommended that selected variables should be based on either previous research or clinical reasoning, so that excluding variables because of missing data are eliminated and consistent data collection is maintained [30]. Additionally, the model showed great discrimination of the severity of COVID-19, with an AUC of 0.939.

Some limitations of this study should be acknowledged. First, this is a monocentric retrospective study and the study population is relatively small, which could inevitably result in some bias. In the future, multicentric studies should be conducted to verify this model. Second, because of the small sample size, it is difficult to set up a validation cohort to assess the predictive accuracy of our nomogram. However, we used a bootstrap resampling cohort for internal validation. Moreover, the calibration curve showed a great agreement between the prediction of the nomogram and the observations in the primary cohort.

## Conclusion

We reported a simple and easy-to-use model (nomogram) that is based on the initial clinical and chest CT characteristics to predict the severity of COVID-19 and with a high discrimination rate of 93.9%. This nomogram can help clinicians to easily and quickly identify patients who may progress to a severe/critical clinical type at the beginning of admission, and timely choose available treatments, thus, helping clinicians to be better prepared for the severe condition of COVID-19. Additionally, under such a severe epidemic, early intervention may alter the outcomes for patients, who may develop to severe conditions.

**Summary points**

- Because of the bad prognosis of severe/critical COVID-19, it is of vital importance to predict the severity of COVID-19 on the initial admission.
- Some studies only focus on the differences between common type and severe/critical type COVID-19; other studies have built models to predict the severity of COVID-19 depending on deep-learning algorithms, which is more difficult to build and not available for all institutions.
- In this study, we build a simple and easy-to-use nomogram based on the clinical and radiological characteristics of COVID-19, to predict the severity of COVID-19 on the initial admission.
- The nomogram consists of five variables including age, neutrophil count, creatinine, procalcitonin and number of involved lung segments, showing a great discrimination rate of 93.9%.
- This model can help clinicians to be better prepared for the severe condition of COVID-19 and choose suitable and timely treatment for patients with severe/critical COVID-19.
